# Adjuvant Chemoradiation in Resected Biliary Adenocarcinoma: Evaluation of SWOG S0809 with a Large National Database

**DOI:** 10.1245/s10434-024-15117-y

**Published:** 2024-03-05

**Authors:** Dana A. Dominguez, Paul Wong, Yi-Jen Chen, Gagandeep P. Singh, Yuman Fong, Daneng Li, Philip H. G. Ituarte, Laleh G. Melstrom

**Affiliations:** 1 Department of Surgical Oncology, City of Hope National Medical Center, Duarte, CA USA; 2 School of Medicine, University of California, San Francisco, San Francisco, CA USA; 3 Department of Radiation Oncology, City of Hope National Medical Center, Duarte, CA USA; 4 Department of Medical Oncology, City of Hope National Medical Center, Duarte, CA USA

**Keywords:** Chemoradiation, SWOG 0809, Bile duct cancer, Gallbladder cancer, Cholangiocarcinoma

## Abstract

**Background:**

There is a paucity of evidence supporting the use of adjuvant radiation therapy in resected biliary cancer. Supporting evidence for use comes mainly from the small SWOG S0809 trial, which demonstrated an overall median survival of 35 months. We aimed to use a large national database to evaluate the use of adjuvant chemoradiation in resected extrahepatic bile duct and gallbladder cancer.

**Methods:**

Using the National Cancer Database, we selected patients from 2004 to 2017 with pT2-4, pN0-1, M0 extrahepatic bile duct or gallbladder adenocarcinoma with either R0 or R1 resection margins, and examined factors associated with overall survival (OS). We examined OS in a cohort of patients mimicking the SWOG S0809 protocol as a large validation cohort. Lastly, we compared patients who received chemotherapy only with patients who received adjuvant chemotherapy and radiation using entropy balancing propensity score matching.

**Results:**

Overall, 4997 patients with gallbladder or extrahepatic bile duct adenocarcinoma with available survival information meeting the SWOG S0809 criteria were selected, 469 of whom received both adjuvant chemotherapy and radiotherapy. Median OS in patients undergoing chemoradiation was 36.9 months, and was not different between primary sites (*p* = 0.841). In a propensity score matched cohort, receipt of adjuvant chemoradiation had a survival benefit compared with adjuvant chemotherapy only (hazard ratio 0.86, 95% confidence interval 0.77–0.95; *p* = 0.004).

**Conclusion:**

Using a large national database, we support the findings of SWOG S0809 with a similar median OS in patients receiving chemoradiation. These data further support the consideration of adjuvant multimodal therapy in resected biliary cancers.

Adenocarcinoma of the biliary tree can occur anywhere along the intrahepatic, perihilar, and distal bile ducts, as well as in the gallbladder. Subtypes of biliary cancers share a similar cellular lineage from bile duct epithelium, however their respective anatomic locations have considerable implications on recurrence and outcomes.^1,2^ Although surgical resection represents the only possibility of cure, recurrence still occurs in around 80% of patients after curative-intent resections.^3^

The rarity of these tumors makes robust studies for these disease sites particularly difficult. Multiple disease sites are often included and this leads to challenges in making comparisons between trials. Despite these challenges, there has been success in demonstrating the benefit of adjuvant chemotherapy in patients with resected biliary cancers.^4–6^ Evidence for the benefit of adjuvant chemoradiation is much more sparse.^7^ The SWOG S0809 phase II, single-arm trial is the most robust examination of this treatment modality, demonstrating a 2-year survival of the entire cohort of 65%, which was significantly improved compared with historical controls.^8^

In this study we sought to support the findings of the SWOG S0809 trial assessing oncologic benefits of adjuvant chemoradiation in extrahepatic cholangiocarcinoma and gallbladder cancer using a large national database, as well as compare them with patients receiving adjuvant chemotherapy only using a propensity matched cohort.

## Methods

### Data Source

The National Cancer Database participant user files (NCDB PUFs) were the source of all data in our study. The NCDB is a nationwide repository of de-identified patient data related to cancer care metrics and outcomes in the United States (US) derived from the submissions of over 1500 Commission on Cancer (CoC)-accredited programs. The NCDB captures over 70% of new cancer diagnoses in the US per year. The CoC is a multidisciplinary association maintained by the American College of Surgeons and the American Cancer Society that accredits US hospitals based on various aspects of cancer care. Due to our study’s inclusion of only de-identified data, it was exempt from Institutional Review Board review.

### Selection Criteria

Patients with surgically resected, pathologically confirmed extrahepatic and gallbladder cancer as a single primary diagnosed from 2004 to 2017 were identified using the International Classification of Disease for Oncology, 3rd edition (ICD-O-3) topography codes C23.9 and C24.0. Intrahepatic and ampullary tumors were excluded. We then selected patients with adenocarcinoma with ICD-O-3 morphology codes for adenocarcinoma 8140 and 8160. The majority of patients included were prior to the inclusion of Collaborative Site-Specific Factor 25 allowing for more granular detail of location on the bile duct, meaning that extrahepatic bile duct and hilar tumors are included together under C24.0. We used the SWOG S0809 selection criteria to select patients with pT2-4, N0-1, M0 who underwent complete resection, excluding patients with an R2 resection. We used this cohort of patients who underwent surgery regardless of receipt of adjuvant therapy (*n* = 4997) to examine factors associated with improved overall survival (OS) (Tables 1 and 2). Next, to examine the effect of the use of adjuvant radiation, we created a ‘SWOG-like’ cohort by further selecting patients who received both adjuvant radiation and multi-agent chemotherapy (*n* = 469) (Table 3). Radiation and chemotherapy sequencing was defined using the ‘RX_SUMM_SURGRAD_SEQ’ and ‘RX_SUMM_CHEMO’ variables. Lastly, in an attempt to reduce interference from known confounders, we created a propensity matched cohort using the same selection criteria as the *n* = 4997 cohort, but only selecting patients who received adjuvant chemotherapy only (regardless of the number of chemotherapy agents), and compared them with those who received adjuvant chemotherapy and radiation (*n* = 2303) (Table 4).

**Table 1:** Demographics of patients who underwent surgical resection regardless of adjuvant therapy (*n* = 4997)

		*n*	%
Age at diagnosis (median (IQR))		68	(60–76)
Sex	Male	2064	41.3
	Female	2933	58.7
Race	White	3948	79.0
	African-American	607	12.1
	Asian	206	4.1
	Other/unknown	236	4.7
Insurance status	Uninsured	176	3.5
	Private/managed care	1641	32.8
	Medicaid	331	6.6
	Medicare	2727	54.6
	Other/unknown	122	2.4
Charlson–Deyo comorbidity score	0	3464	69.3
	1	1095	21.9
	≥2	438	8.8
ECC vs. GBC	Extrahepatic cholangiocarcinoma	1716	34.3
	Gallbladder adenocarcinoma	3281	65.7
Combined pT stage AJCC 7	pT2	2429	48.6
	pT3	2432	48.7
	pT4	136	2.7
Combined pN stage AJCC 7	pN0	2701	54.1
	pN1	2296	45.9
Grade	Well differentiated	580	12.2
	Moderately differentiated	2416	50.9
	Poorly/undifferentiated	1751	36.9
Margin status	R0	4286	85.8
	R1	711	14.2
Lymphovascular invasion	Absent	1894	46.8
	Present	1505	37.2
	Unknown	650	16.1
Adjuvant radiation	No	3677	73.6
	Yes	1320	26.4
Adjuvant chemotherapy	No	2322	46.5
	Yes	2675	53.5
Adjuvant chemoradiation	No	4528	90.6
	Yes	469	9.4
Chemotherapy agents	No chemotherapy	2322	46.5
	Single-agent chemotherapy	1230	24.6
	Multi-agent chemotherapy	1075	21.5
	Unknown chemotherapy agents	370	7.4

ECC extrahepatic cholangiocarcinoma, GBC gallbladder cancer, AJCC American Joint Committee on Cancer

**Table 2:** Cox regression model of factors associated with survival in patients who underwent resection regardless of adjuvant therapy (*n* = 4997)

		Univariable analysis					Multivariable analysis			
				95% CI				95% CI		
Patient characteristics		*N*	HR	Lower	Upper	*p*-value	HR	Lower	Upper	*p*-value
Age		4996	1.02	1.02	1.03	< 0.001	1.02	1.01	1.02	< 0.001
Sex	Male	2064	Ref	–	–	–	Ref	–	–	–
	Female	2932	0.89	0.83	0.95	<0.001	0.94	0.86	1.03	0.167
Race	White	3948	Ref	–	–	–	Ref	–	–	–
	African-American	606	0.93	0.83	1.03	0.18	1.03	0.91	1.18	0.615
	Asian	206	0.84	0.71	1.01	0.063	0.89	0.72	1.09	0.244
	Other/unknown	236	0.67	0.55	0.80	< 0.001	0.82	0.67	1.01	0.063
Insurance status	Uninsured	176	Ref	–	–	–	Ref	–	–	–
	Private/managed care	1641	0.79	0.65	0.97	0.02	0.72	0.57	0.90	0.005
	Medicaid	331	0.86	0.68	1.10	0.234	0.81	0.61	1.06	0.125
	Medicare	2726	1.19	0.98	1.45	0.08	0.85	0.67	1.07	0.16
	Other/unknown	122	1.00	0.75	1.34	0.998	0.75	0.53	1.06	0.102
Charlson–Deyo comorbidity score	None	3463	Ref	–	–	–	Ref	–	–	–
	1	1095	1.09	1.01	1.19	0.03	1.08	0.98	1.19	0.132
	≥2	438	1.30	1.16	1.47	< 0.001	1.23	1.07	1.41	0.003
Site	ECC	1716	Ref	–	–	–	Ref	–	–	–
	GBC	3280	0.89	0.83	0.96	0.002	1.08	0.98	1.19	0.123
pT	pT2	2428	Ref	–	–	–	Ref	–	–	–
	pT3	2432	1.73	1.62	1.86	< 0.001	1.57	1.43	1.72	< 0.001
	pT4	136	2.52	2.08	3.05	< 0.001	2.07	1.59	2.68	< 0.001
pN	N0	2700	Ref	–	–	–	Ref	–	–	–
	N1	2296	1.68	1.57	1.80	< 0.001	1.62	1.48	1.77	< 0.001
Grade	Well differentiated	580	Ref	–	–	–	Ref	–	–	–
	Moderately differentiated	2415	1.29	1.15	1.46	< 0.001	1.16	1.01	1.33	0.034
	Poorly/undifferentiated	1751	1.77	1.57	2.01	< 0.001	1.52	1.32	1.76	< 0.001
LVI	Absent	1894	Ref	–	–	–	Ref	–	–	–
	Present	1505	1.57	1.44	1.71	< 0.001	1.24	1.13	1.36	< 0.001
	Unknown	650	1.15	1.02	1.29	0.018	1.06	0.94	1.20	0.357
Margin status	R0	4285	Ref	–	–	–	Ref	–	–	–
	R1	711	2.09	1.92	2.29	< 0.001	1.93	1.73	2.14	< 0.001
Adjuvant chemotherapy	No	2322	Ref	–	–	–	Ref	–	–	–
	Yes	2675	0.83	0.78	0.89	0.06	0.76	0.69	0.84	< 0.001
Adjuvant radiotherapy	No	3677	Ref	–	–	–	Ref	–	–	–
	Yes	1319	0.85	0.79	0.92	< 0.001	0.86	0.77	0.95	0.003
‘SWOG-like’ chemo/radiation	No	4527	Ref	–	–	–	–	–	–	–
	Yes	469	0.81	0.72	0.91	< 0.001	–	–	–	–

HR hazard ratio, CI confidence interval, Ref reference, ECC extrahepatic cholangiocarcinoma, GBC gallbladder cancer, LVI lymphovascular invasion

**Table 3:** Demographics of the ‘SWOG-like’ cohort who received adjuvant chemoradiation (*n* = 469)

		*n*	%
Age at diagnosis (median (IQR))		63	(56–69)
Sex	Male	201	42.9
	Female	268	57.1
Race	White	376	80.2
	African-American	55	11.7
	Asian	20	4.3
	Other/unknown	18	3.8
Insurance status	Uninsured	13	2.8
	Private/managed care	221	47.1
	Medicaid	33	7.0
	Medicare	185	39.4
	Other/unknown	17	3.6
Charlson–Deyo comorbidity score	0	338	72.1
	1	101	21.5
	≥2	30	6.4
ECC vs. GBC	Extrahepatic cholangiocarcinoma	211	45.0
	Gallbladder adenocarcinoma	258	55.0
Combined pT stage AJCC 7	pT2	177	37.7
	pT3	281	59.9
	pT4	11	2.3
Combined pN stage AJCC 7	pN0	146	31.1
	pN1	323	68.9
Grade	Well differentiated	44	9.9
	Moderately differentiated	232	52.4
	Poorly/undifferentiated	167	37.7
Margin status	R0	387	82.5
	R1	82	17.5
Lymphovascular invasion	Absent	162	37.9
	Present	197	46.0
	Unknown	69	16.1
Adjuvant chemoradiation	No	0	0.0
	Yes	469	100.0

IQR interquartile range, ECC extrahepatic cholangiocarcinoma, GBC gallbladder cancer, AJCC American Joint Committee on Cancer

**Table 4:** Pre- and post-balancing values for treatment and control groups for the matched cohort

	Treatment			Control		
	Mean	Variance	Skewness	Mean	Variance	Skewness
*Before weighting*						
Age	63.79	108.3	−0.3089	64.85	110.3	−0.4342
Comorbidity	0.3461	0.3644	1.547	0.3713	0.3909	1.46
Pathological T stage	2.618	0.2834	−0.01219	2.637	0.3057	0.1027
Pathological N stage	0.5963	0.2409	−0.3927	0.5829	0.2433	−0.3363
LVI	3.06	15.49	0.8233	2.509	13.2	1.194
Margins	0.1857	0.1513	1.616	0.1218	0.107	2.313
*After weighting*						
Age	63.79	108.3	−0.3089	63.79	108.3	−0.3401
Comorbidity	0.3461	0.3644	1.547	0.3461	0.3644	1.547
Pathological T stage	2.618	0.2834	−0.01219	2.618	0.2834	−0.01201
Pathological N stage	0.5963	0.2409	−0.3927	0.5963	0.2409	−0.3927
LVI	3.06	15.49	0.8233	3.06	15.49	0.8233
Margins	0.1857	0.1513	1.616	0.1857	0.1513	1.617

LVI lymphovascular invasion

### Statistical Analysis

We performed univariable analysis to identify clinicopathologic factors of patients with resected extrahepatic and gallbladder cancer. Continuous variables were reported as median and interquartile range (IQR), while categorical variables were described using counts and percentages. We performed univariable and multivariable analysis using a Cox proportional hazards model; factors with a *p*-value < 0.10 on univariable analysis were included in the multivariable model. OS analysis was performed using the Kaplan–Meier method and log-rank test to examine survival stratified by various categorical variables, including site (extrahepatic vs. gallbladder), nodal status, and R status. Lastly, a chart was created to analyze trends in the use of a ‘SWOG-like’ protocol for adjuvant therapy, as a percentage of all patients who underwent resection (*n* = 4997) per year. Statistical analyses were performed using IBM SPSS Statistics for Windows, version 25.0 (IBM Corporation, Armonk, NY, USA). Statistical significance was defined by a two-tailed *p*-value < 0.05.

For propensity score matched analysis, variables potentially associated with treatment group were analyzed by applying Student’s t-test for continuous variables (i.e., age) or the Chi-square test for categorical variables. Variables significantly associated with treatment group included age, Charlson comorbidity score, pathological T stage, pathological N stage, and resection margins. Sex, race, and insurance were not associated with treatment group and were excluded from additional analyses. Entropy balancing was applied to create propensity score matching (PSM) of variables for age, comorbidity, pathological T stage, pathological N stage, lymphovascular invasion, and resection margins. “Entropy balancing relies on a maximum entropy reweighting scheme that calibrates unit weights so that the reweighted treatment and control group satisfy a potentially large set of prespecified balance conditions that incorporate information about known sample moments”.^9^ Specifically, the ‘sample moments’ are the mean, variance, and skewness, and the balance conditions are covariates associated with both the treatment and control groups. Entropy-balanced weights ensure that the values for mean, variance, and skewness are identical for both the treatment and control groups. Unlike coarsened exact matching or other PSM methods, entropy balancing may be achieved without discarding any cases. Following this step, a multivariable Cox proportional hazard model was created that included the matched variables in addition to tumor site, which was associated with survival time in univariable Cox proportional hazard analysis. Entropy balancing and Cox proportional hazard models were performed using Stata MP version 14.2 (StataCorp LLC, College Station, TX, USA). Stata’s ‘ebalance’ program was used for entropy balancing.^10^

## Results

### Patient Demographics

In patients who underwent resection, regardless of receipt of adjuvant therapy (*n* = 4997), the majority had a gallbladder primary tumor (*n* = 3281, 65.7%), compared with an extrahepatic bile duct primary (*n* = 1716, 34.3%). In regard to patient characteristics, the majority of patients were female (*n* = 2933, 58.7%), White (*n* = 3948, 79.0%), and insured (*n* = 4821, 96.5%), with a low Charlson–Deyo comorbidity index. Tumors were mostly T2 (*n* = 2429, 48.6%) and T3 (*n* = 2432, 48.7%) and well-balanced with respect to nodal status (N0; *n* = 2701, 54.1%). In regard to adjuvant therapy, 53.5% of patients received at least single-agent adjuvant chemotherapy. Most patients who received adjuvant radiation therapy also received chemotherapy (*n* = 1233, 93.4%) (Table 1).

On multivariable analysis of factors associated with survival in this cohort, older age, Charlson Deyo score ≥ 2, pT stage, pN stage, higher grade, presence of LVI, and positive margin were all significantly associated with poor survival, while private insurance status and receipt of either adjuvant chemotherapy or radiation were associated with improved OS. The use of a ‘SWOG-like’ adjuvant regimen (multi-agent chemotherapy and radiation) was associated with a survival advantage on univariable analysis (HR 0.81 95% confidence interval [CI] 0.72–0.91, *p* < 0.001) (Table 2).

### Survival Analysis in the ‘SWOG-Like’ Cohort

**Fig. 1:**
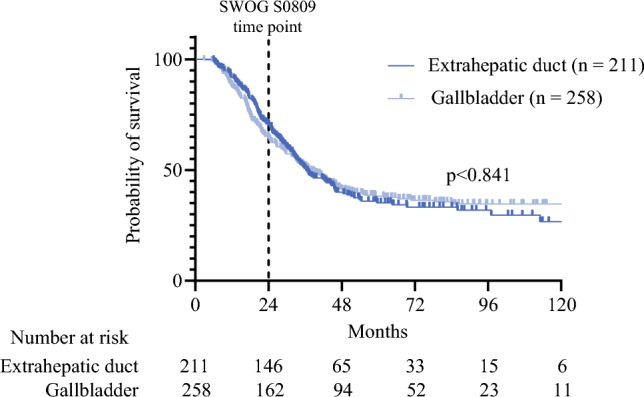
Overall survival of the ‘SWOG-like’ cohort stratified by primary tumor site

**Fig. 2:**
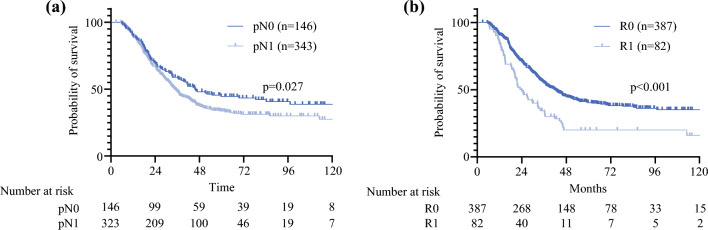
Overall survival in the ‘SWOG-like’ cohort stratified by **a** nodal status and **b** resection margin status

Patients who received a ‘SWOG-like’ regimen (*n* = 469) had more advanced pT stage (pT3: *n* = 281, 59.9%) and more were node-positive (pN1: *n* = 323, 68.9%). Most patients who received this regimen had an R0 resection (*n* = 387, 82.5%) (Table 3). OS in patients receiving this course of adjuvant therapy had a median OS of 36.9 months, with 65.6% of patients alive at 2 years. There was no difference in OS when stratified by primary tumor site (*p* = 0.841) (Fig. 1); however, when stratified by nodal status, while there was no difference in percentage survival at the 2-year timepoint (67.8% vs. 64.7%), median OS was significantly better in patients with negative nodes (45.7 vs. 35.0 months, *p* = 0.027) (Fig. 2a). Patients with R0 resection margins also had significantly better median OS than patients who underwent an R1 resection (41.8 vs. 24.1 months, *p* < 0.001) (Fig. 2b).

### Survival Analysis in Propensity Matched Cohort

**Fig. 3:**
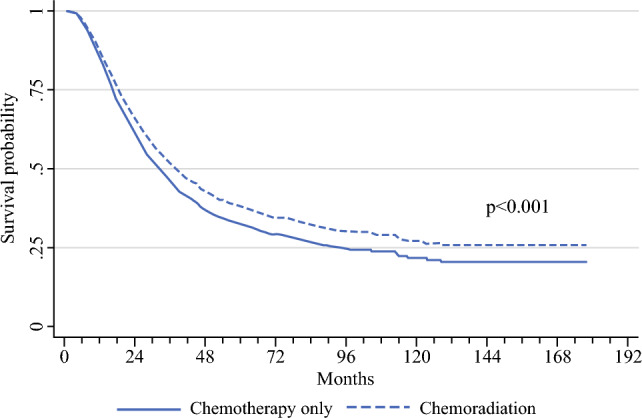
Multivariable Cox proportional hazards model of propensity matched cohorts comparing patients who received chemotherapy only with those who received chemotherapy and radiation (*n* = 2303)

The pre- and post-balancing values for the treatment and control groups on three statistical parameters are presented in Table 4. The balanced model hazard ratios (HRs) are presented in Table 5. Age, gallbladder primary site, T stage, N stage, presence of LVI, and a positive margin were all associated with worse OS, while the receipt of chemoradiation was associated with improved survival (HR 0.86, 95% CI 0.77–0.95, *p* = 0.004). Patients who received adjuvant chemoradiation had a longer median survival compared with adjuvant chemotherapy only, regardless of number of chemotherapy agents used (36.7 months vs. 31.7 months; *p* < 0.025) (Fig. 3).

**Table 5:** Multivariable Cox proportional hazard model for the propensity matched cohort comparing patients who received chemotherapy only versus chemoradiation (*n* = 2303)

Variable		HR (95% CI)	*p*-value
Age		1.02 (1.01–1.02)	< 0.001
Site	Extrahepatic (ref)	–	–
	Gallbladder	1.19 (1.05–1.34)	0.005
Pathological T stage	T2 (ref)	–	–
	T3	1.60 (1.41–1.81)	< 0.001
	T4	2.22 (1.62–3.03)	< 0.001
Pathological N stage	N0 (ref)	–	–
	N1	1.48 (1.32–1.67)	< 0.001
LVI	Absent (ref)	–	–
	Present	1.29 (1.14–1.46)	< 0.001
	Unknown	1.11 (0.96–1.28)	0.144
Margins	R0 (ref)	–	–
	R1	1.61 (1.39–1.86)	< 0.001
Chemoradiation	Control (ref)	–	–
	Treatment	0.86 (0.77–0.95)	0.004

HR hazard ratio, CI confidence interval, LVI lymphovascular invasion, ref reference

## Discussion

Using strictly selected cohorts of patients who underwent surgical resection of extrahepatic or gallbladder adenocarcinoma, we were able to validate the findings of the SWOG S0809 trial. Our data successfully reproduced OS at 2 years, a benefit that was sustained with longer-term follow-up.

Studies of recurrence patterns in extrahepatic bile duct cholangiocarcinoma and gallbladder cancer illustrate the potential for benefit of using adjuvant radiation for improved local control, with rates of locoregional-only recurrence of 39–51% and 15.8%, respectively.^11,12^ However, while a number of large prospective randomized controlled trials exist supporting adjuvant chemotherapy,^4,6,13^ support for the use of adjuvant chemoradiation in extrahepatic bile duct cancers remains scarce. The SWOG S0809 trial remains the only prospective trial evaluating the addition of radiation in the adjuvant setting. Its phase II and single-arm nature, as well as an overall small cohort of only 79 patients, limited the overall power of the study, however it did demonstrate significant improvement in OS compared with historical controls.^8^ This trial also helped illustrate the issues that exist when grouping disease sites together within trials that have disparate biology, highlighted by the differences in nodal involvement (extrahepatic cholangiocarcinoma 75.6% vs. gallbladder cancer 24.4%), as well as a vastly different rate of distant metastasis between hilar and distal cholangiocarcinoma (8% vs. 35%).^14,15^ These significant differences illustrate the potential differences in disease biology between these disease sites, one of the most significant issues facing trial design for studying relatively rare tumors. Of course, as more disease sites are included, it is easier to accrue a sufficient number of patients to detect a difference, with the trade-off of potentially less clear results from a more heterogeneous cohort. In that trial, there was no difference in 2-year OS between gallbladder and extrahepatic primary sites (53% vs. 68%; *p* = 0.87), a finding that was consistent with our findings, with a combined median OS of 37 months. Interestingly, in a re-analysis of the SWOG trial patients that focused on nodal status, Gholami et al. found no significant difference in 2-year OS between N0 and N+ patients (70.6% vs. 60.9%; *p* = 0.11);^14^ however, in our data, the curves diverge significantly only after the 2-year timepoint, likely benefiting from significantly longer-term follow-up. We demonstrate a significant median survival benefit in patients with negative lymph nodes (45.7 vs. 35.0 months; *p* = 0.027). The SWOG S0809 trial also did not detect any difference in survival between patients with an R0 and R1 resection, which was significantly different from findings in our data, which showed a significantly worse median OS in patients with a microscopically positive resection margin (41.8 vs. 24.1 months; *p* < 0.001). This disparity may be attributed in part to the significantly larger sample size in this cohort.

Since the SWOG S0809 study, there have been multiple retrospective cohorts that have supported the use of chemoradiation in the adjuvant setting. In a large cohort of 1475 patients with extrahepatic bile duct cancer, the use of combined radiotherapy followed by chemotherapy was associated with optimal survival outcomes (HR 0.52, 95% CI 0.41–0.68).^16^ Another similar cohort of non-hilar extrahepatic bile duct cancer, as well as a separate cohort of 100 patients with gallbladder cancer, both found that use of adjuvant chemoradiation was an independent prognostic factor of OS (*p* < 0.05).^17,18^ Similarly, in an examination of only high-risk extrahepatic cholangiocarcinoma (R1 and/or N^+^), an analysis of the NCDB found significant survival benefit with adjuvant radiation.^19^ Our findings were consistent with this evidence, not only demonstrating the use of chemoradiation as an independent prognostic factor but also significantly improving survival in a propensity matched cohort of patients.

This study has limitations inherent to all analyses of large databases, including missing data and a lack of recurrence data. Importantly, the NCDB lacks data regarding the specifics of adjuvant therapy, including specific chemotherapy and radiation regimens. We recognize the significant limitations to this fact, and indeed it is possible that patients in either arm received the combination of gemcitabine and oxaliplatin, a regimen that was demonstrated to be no better than surveillance in the PRODIGE 12 trial, which could potentially bias our data in favor of chemoradiation.^20^ However, we feel that it is more likely that the proportion of patients in both arms received a more established regimen with demonstrated efficacy such as single-agent capecitabine, gemcitabine/cisplatin, or gemcitabine/capecitabine. Additionally, all patients included in our study were treated prior to enrollment of the TOPAZ-1 trial, making it unlikely that the addition of immunotherapy significantly influences our data.^21^ While there is some ability to control for the amount of radiation received, data with the variables pertaining to these factors have significant missing data. We attempted to mitigate these limitations as much as possible through a strict selection criteria for the ‘SWOG-like’ cohort, as well as the propensity matched cohort comparing chemotherapy alone regardless of number of agents used, to chemoradiation while controlling for known confounders.

## Conclusion

Using a large national database, we provide further evidence supporting the findings of the SWOG S0809 study, with an overall 2-year survival of 65.6% in patients who received adjuvant radiation in addition to chemotherapy, further highlighting the need for validation in a prospective setting.
